# An Account of a Disease Occasioned by Transplanting a Tooth

**Published:** 1786

**Authors:** William Watson

**Affiliations:** Fellow of the College of Physicians, and Vice-President of the Royal Society


XVII. *An Account of a Disease occasioned by transplanting a Tooth.* By William Watson, *M. D. Fellow of the College of Physicians, and Vice-President of the Royal Society.* Vide *Medical Transactions, published by the College of Physicians in London. Volume the Third,* 8vo. *London,* 1785.

THE subject of this melancholy case was an unmarried lady, aged about twenty-one, and of a delicate habit, but in other respects in perfect health. One of the incisores of the upper jaw happening to become carious, she determined to have it removed, and replaced by a sound tooth. This was accordingly done, the transplanted tooth being taken from the mouth of an apparently-healthy person. The

The new tooth faſtened ſo well, that the dentiſt, on account of its firm ſtate, removed the ſilk, employed to faſten it to the neighbouring teeth, ſooner than he had been accuſtomed to do: but although the tooth faſtened, and continued firm for about a month, the patient obſerved that her mouth felt tender all that time. This, however, ſhe conſidered as a neceſſary conſequence of the operation.

At the end of about a month, her mouth became very painful. The upper gums were at firſt inflamed and enlarged, but afterwards became diſcoloured and ulcerated. The ulceration, Dr. Watſon obſerves, ſpread ſo faſt, that the gums of the upper jaw were corroded away, ſo as to leave the alveoli bare, and, before another month had elapſed, the ulceration, he adds, occupied the whole ſpace under the upper lip between the teeth and the noſe, and extended likewiſe to the cheeks and throat, which were corroded by large, deep, and fœtid ulcers; the attending to the inceſſant diſcharge from which deprived her of ſleep, while the ſoreneſs of the parts prevented her taking ſufficient nouriſhment.

About this time her face and other parts of her body began to be covered with blotches,

ſeveral

ſeveral of which ulcerated, and, by the irrita-tion they occaſioned, heightened a fever, under which ſhe laboured, to ſuch a degree, that her death was expected.

It was in this ſtate of the diſeaſe that Dr. Watſon's aſſiſtance was deſired. She had, for ſome time, he tells us, been under the care of the dentiſt; but when her complaints became more alarming, an able and experienced ſur-geon (Mr. Hunter, as we now learn from his own account* of the caſe) was conſulted, who directed remedies for the ulcerated mouth, and gave her bark freely, both in decoction and ſubſtance, together with anodynes occaſionally; notwithſtanding which, the ulcerations, we are told, continued daily to grow worſe.

In this depraved ſtate of her general health Dr. Watſon recommended aſſes milk, and a li-beral uſe of bark, combined with myrrh; but as the weak ſtate of her ſtomach would not al-low her to take a ſufficient quantity of theſe re-medies to produce any good effect, he thought it right, at the end of four or five days, to have recourſe to mercury; and accordingly ſhe took a pill, containing two grains of calomel, once

* See page 211.

or twice a day, till ſhe had taken about fourteen pills, when the ſtate of her bowels made it neceſſary to deſiſt. Even theſe ſmall doſes of mercury, however, were obſerved to be of great uſe, as the ulceration of her mouth was leſs painful, and did not ſpread while ſhe was taking them; and the blotches on her face and body grew paler, and, ſuch of them as had ulcerated, healed apace.

As the ſtate of her bowels would allow no more mercury to be adminiſtered by the mouth, ſmall doſes of *unguent. coerul. fort.* were now directed to be rubbed into her legs and thighs twice a day; but this treatment was likewiſe obliged to be diſcontinued in about ten or twelve days, on account of the irritable ſtate of her bowels; but at this time, the author obſerves, the blotches had all diſappeared, and the ulcerations on her face and body were completely healed, and thoſe of her mouth nearly ſo.

The caſe now began to aſſume all the appearances of a confirmed hectic fever; and the patient, reduced by her long-continued ſufferings, and her want of ſleep and nouriſhment, was every night oppreſſed with colliquative ſweats. Theſe were in ſome degree, however, leſſened by the judicious mode of treatment recommended commended by the author, and ſhe recovered a ſufficient degree of ſtrength to be removed to her uſual place of reſidence, which was about eighty miles from London; but upon her return into the country, her ſtrength gradually leſſened, till death put an end to her ſufferings.

In his remarks on the caſe, Dr. Watſon obſerves, that the progreſs of this putrid diſeaſe not being impeded by the moſt powerful antiſeptics, in liberal doſes, and the circumſtance of its having given way to mercurials, even in ſmall ones, cannot but furniſh room to ſuggeſt, that the taint was truly venereal. But the dentiſt who performed the operation aſſured him, that the perſons from whom the teeth to be tranſplanted are taken, are always examined by others, as well as by himſelf, with regard to the ſtate of their health; and that, in the preſent caſe, the young woman from whom the tooth was taken had been inſpected by a very eminent ſurgeon with reſpect to the ſtate both of her tonſils and pudendum, and was found to be without the leaſt appearance of diſeaſe. But admitting that the pudendum had been affected, the gums and teeth, Dr. Watſon remarks, appeared to be perfectly ſound, and, under that circumſtance, he thinks it is difficult to conceive how mifchief could arife from thence. Admitting, however, for the fake of argument, but which, the author obferves, he does not believe, that the young lady, the fubject of this paper, had received the infection in the ufual way, it would, he thinks, not have had fo violent an effect on the mouth, and have left, which was really the cafe, the pudendum free. "In whatever manner, therefore," obferves the learned author, "we fearch for the "caufe of this malady, difficulties, to me, at "leaft, infurmountable, prefent themfelves." For this reafon, he has thought it expedient, he tells us, to lay down the whole hiftory of the cafe, that perfons converfant in matters of this fort may themfelves deduce fuch conclufions from it as they apprehend will carry the greateft degree of probability of their being well founded.

